# Pollen Source Richness May Be a Poor Predictor of Bumblebee (*Bombus terrestris*) Colony Growth

**DOI:** 10.3389/finsc.2021.741349

**Published:** 2021-12-01

**Authors:** Cecylia M. Watrobska, Ana Ramos Rodrigues, Andres N. Arce, Jessica Clarke, Richard J. Gill

**Affiliations:** Department of Life Sciences, Imperial College London, Silwood Park Campus, London, United Kingdom

**Keywords:** social bees, workers, pupae, land use, monoculture, agriculture, insect pollinator, foraging

## Abstract

Agricultural intensification has drastically altered foraging landscapes for bees, with large-scale crop monocultures associated with floral diversity loss. Research on bumblebees and honeybees has shown individuals feeding on pollen from a low richness of floral sources can experience negative impacts on health and longevity relative to higher pollen source richness of similar protein concentrations. Florally rich landscapes are thus generally assumed to better support social bees. Yet, little is known about whether the effects of reduced pollen source richness can be mitigated by feeding on pollen with higher crude protein concentration, and importantly how variation in diet affects whole colony growth, rearing decisions and sexual production. Studying queen-right bumblebee (*Bombus terrestris*) colonies, we monitored colony development under a polyfloral pollen diet or a monofloral pollen diet with 1.5–1.8 times higher crude protein concentration. Over 6 weeks, we found monofloral colonies performed better for all measures, with no apparent long-term effects on colony mass or worker production, and a higher number of pupae in monofloral colonies at the end of the experiment. Unexpectedly, polyfloral colonies showed higher mortality, and little evidence of any strategy to counteract the effects of reduced protein; with fewer and lower mass workers being reared, and males showing a similar trend. Our findings (i) provide well-needed daily growth dynamics of queenright colonies under varied diets, and (ii) support the view that pollen protein content in the foraging landscape rather than floral species richness *per se* is likely a key driver of colony health and success.

## Introduction

Bees are essential insect pollinators of many wild flowers and crops, making reported declines an issue of global importance (1–7). The emergence of agricultural land-use has been implicated as a contributing driver [e.g., (8–11)]. Large scale crop monocultures leading to fragmentation and loss of wild floral resources (12, 13) are thought to have degraded the “nutritional landscape” by lowering nectar and pollen availability to bees (14–16). Increased cultivation of pollinator-dependent crops since the 1960's may therefore be encouraging news by helping to nutritionally subsidize wildflower losses (17, 18). However, having large swathes of single plant species/varieties can reduce the diversity of florally sourced nectar and pollen available (19) leading to agri-environment schemes promoting management of diverse floral resources to support bees (1, 20, 21). But, is focusing on floral diversity the most important criterion when informing such schemes? For instance, it may also be important to understand how the amount and/or quality of nectar and pollen provisioned in these floral habitats contributes to bee reproductive success (22–25).

For bumblebees, floral pollen is the exclusive protein source needed for colony growth (26), influencing the number and size (mass) of reared individuals, both of which have a positive feedback on future pollen income (27, 28). Under natural settings, however, there is a limit to the amount of pollen that can be brought back to the colony because workers are restricted by foraging ranges, weather conditions and temporal food resource gaps (1, 29–31). Landscapes dominated by floral species possessing pollen of high crude protein may therefore be beneficial and help to counteract such constraints. Indeed, studies of bumblebee micro-colonies (a subset of colony workers kept together without a queen) have shown that pollen from one plant species can be associated with increased growth compared with provision from another (24, 32–39). However, feeding on pollen from a monofloral source may come at a cost of losing pollen nutritional diversity (35, 40–42), with any reduction in floral species richness potentially leading to deficiencies in essential nutrients (19).

An increase in the diversity of florally sourced pollen has been shown to be associated with improved individual bee condition, such as longevity (43–45) and immune capacity (41, 46–48), as well as benefitting a set of micro-colony parameters (e.g., egg production and larval weight) (41, 49). However, when studying the effect of diet on bumblebee colony growth rates, we still have a limited understanding as to how the potential benefits of feeding on pollen from different floral species may be mediated by crude protein concentration (24, 38). Insights gained by such experimentation contribute to informing management practices when advising on floral composition of habitats to support bumblebees. A first step is to better understand comparative colony responses to provision of pollen of relative high crude protein content but of low floral source diversity vs. provision of pollen of relative lower content but of higher diversity. Whilst pollen diversity could lead to increased worker longevity, we predict that feeding on pollen of lower crude protein content would lead to fewer and/or smaller individuals being reared; but empirical data on number-mass responses in bumblebees is limited (50). To address this requires monitoring of the day-by-day growth dynamics of bumblebee colonies under different diets (37, 51, 52). To date, however, there is also a surprisingly limited amount of data on the relative long term daily dynamics of colony growth (27).

Here we compared colony growth rates over 6 weeks in queen-right bumblebee *Bombus terrestris* colonies provisioned with a polyfloral pollen diet vs. colonies provisioned with a monofloral pollen diet with a relatively higher crude protein concentration. By meticulously monitoring all newly eclosed bees and individual deaths we studied colony growth strategies in response to diet by looking if sequential generations of workers responded differently between the monofloral and polyfloral diet to investigate possible cohort lag effects. We further investigated whether the monofloral diet had long-term impacts by: (i) impacting the later stages of colony development; (ii) influencing colony decisions on the number-mass trade-off when rearing individuals; and (iii) altering mortality rate. Starting with small established queen-right colonies, this experiment measured colony food consumption, colony weight gain, worker and male production, mass of reared individuals, worker mortality, and number of pupae at the end of the 6-week period.

## Methods

### Bumblebee Colonies

Twenty-four colonies were ordered from the commercial supplier Biobest NV (Belgium) and distributed by Agralan Ltd (UK). Colonies were housed inside a self-contained and ventilated plastic nest box (25 × 20 × 13 cm) for the 6-week experiment (42 days). On arrival the sugar solution reservoir and pollen patty provided with the colonies were removed, and all colonies were placed in an environmentally controlled room (23°C, 60% humidity) under continual red light. Twenty-four hours prior to start of the experiment (also 24 h after arrival) each colony was provisioned with a feeder containing 25 mL of 40% sucrose solution, checked for an active queen, and the number of pupae and workers counted.

Two experimental replicates (ERs) were conducted: (i) for ER1, colonies arrived at the approximate size requested with monofloral assigned colonies (*n* = 6 colonies) having a mean (±s.e.m.) of 34.2 ± 3.9 pupae and 24.7 ± 2.0 workers, and polyfloral colonies (*n* = 6) having 26.8 ± 2.8 pupae and 22.8 ± 1.2 workers; (ii) for ER2, colonies arrived slightly larger than requested with monofloral colonies (*n* = 6) having 42.2 ± 5.7 pupae and 47.0 ± 4.8 workers, and polyfloral colonies (*n* = 6) having 37.2 ± 7.9 pupae and 50.5 ± 1.6 workers. Therefore, workers from 11 of the ER2 colonies were culled (random removal) to reduce worker number per colony to 35 (size of the smallest colony; Supplementary Table 1). For each ER, colonies were assigned to treatments by ranking first the number of workers present in the colony on arrival (prior to culling for ER2), which was then coupled with a count estimate of colony pupal number. The sum of these two ranks was then determined with each colony paired with its closest consecutive rank and assigned randomly to either the monofloral or polyfloral treatment. We found no significant difference in worker numbers between monofloral and polyfloral assigned colonies (GLM: workers: *z* = 0.34, *p* = 0.74) or initial colony mass (LM: t = 1.38, *p* = 0.18). Based on our estimated pupal counts there was however a significantly lower number of pupae in polyfloral colonies (mean Δ = −16%; GLM: *z* = −2.55, *p* = 0.011; Supplementary Table 2), which was considered when running our statistical analyses.

### Feeding Regime and Pollen Diets

During the experiment, colonies were provided 40% sucrose solution in a gravity feeder alongside the respective pollen diet inside a 55 mm diameter (12 mm deep) Petri dish, three times per week (Monday, Wednesday, and Friday; colonies fed from days 1 to 38). For sucrose and pollen provision, we incremented the standardized volume/mass as the experiment progressed (see Supplementary Table 3 for set amounts). Our choice to provision limited amounts rather than *ad-libitum* was to simulate a more realistic scenario of colonies being constrained by physical access and availability in the field (see Introduction). Honeybee collected pollen (supplied by Agralan Ltd, UK) was used for making the pollen diets and was stored at −20°C. A subsample was taken each time for provisioning, which ensured it had only just thawed before being provided to colonies. Each time the sucrose feeder and pollen diet provisions were replenished, any remaining sucrose solution and pollen were measured to the nearest 0.1 mL and 1 μg, respectively.

Different colors of the supplied honeybee collected pollen pellets indicated pollen was from multiple flower species. Based on an established method (24, 41, 47), we separated pellets that exhibited either a distinct light purple (ER1) or navy (ER2) color. These two colors were chosen because they were highly distinguishable from the other pollen colors, giving us confidence to reliably and consistently pick pollen of the same color. Furthermore, preliminary analysis of pollen content showed a relatively high level of protein content compared to the other pollen types in the mix, with protein content being similar between purple and navy. The polyfloral diet for both ER1 and ER2 consisted of the remaining pollen mixed with the purple or navy pollen constituting 5% (w/w), respectively. Basing the monofloral diet on color inspection assumed that each similarly colored pollen ball was comprised of mostly one species of flowering plant, considering that honeybees are often florally constant during each foraging bout (53). Palynological analysis of each pollen diet to identify richness and composition of the pollen morphotypes, alongside a Bradford assay to measure crude protein content (see Supplementary Methods), provided support to justify our assumptions made above (see section Results).

### Pollen Analysis

Using a compound microscope (Labophot-2, Nikon, Japan) we determined the morphology of pollen grains from subsamples of pollen from each diet (for preparation of samples see SI Methods). Classification of the pollen grain morphotypes was conducted from photographic images produced by fitting a GX-CAM digital camera (GXCam-5, IS500, 5MP; GT Vision Ltd, UK) to the microscope. Images were captured at ×400 magnification at a resolution of 2592 × 1944 pixels, and manually adjusted exposure using the GT Vision software. For each of the four sample spots per pollen diet, we obtained images of five randomly selected microscopic fields-of-view (total = 20 images; each field of view = 218.7 mm^2^). Morphotypes were described using a combination of seven main characters: (i) size; (ii) shape; (iii) thickness and structure of the exine (outer layer); (iv) ornamentation of the exine; (v) observable apertures and the number; (vi) aperture type; and (vii) level of staining (54) (Supplementary Table 4). As morphotype identification progressed, each morphotype was attributed a unique number and added to a reference picture library compiled to aid in classifying all future pollen morphotypes (Supplementary Figure 1). For each morphotype, we counted the number of pollen grains observed across the 20 images. Taking three representative pollen grains per morphotype, we measured the width at the widest point of the grain and took the mean value. To gain a relative proportion of each morphotype per diet, we took morphotype abundance and divided by the mean grain width, to control for many smaller grains taking up the same space as fewer large grains.

### Colony Monitoring

Prior to the start of the experiment all workers per colony were tagged with a unique color and number Opalith tag. Between experimental days 1–35, colonies were checked every day Monday to Friday, and any newly eclosed workers were tagged allowing us to estimate the age of each bee. Bees were not tagged between days 36 and 42, and so any untagged bees found at the end of the experiment were determined to have eclosed during this last week. Tagging involved removing a newly eclosed bee from a colony using forceps, placing inside a marking cage and applying the tag using superglue. The bee was then allowed to rest for 5 min in an individual holding container, after which it was placed back into the colony. Any dead bees found inside the colony during the tagging process were removed, tag (if present) noted and placed inside an individual 2 mL tube and frozen at −20°C.

On day-1 each colony was weighed (AE Adam®, model PGW1502e, accuracy ± 0.01 g) and a repeated measure of colony mass was taken every 7 days, with the mass of the plastic box subtracted. Colony mass therefore constituted all individuals, wax nest, brood and any pollen and/or nectar stores. On day-42 all colonies were sacrificed by placing the colony box in a freezer (−20°C), and after 24 h each colony was dissected and number of workers, gynes (newly produced queens), males (drones) and pupae were counted, and mass of the nest structure weighed. The wet mass per worker was taken, and additionally the mean wet mass per pupae was calculated by taking the total mass and dividing by the total number of individuals. Whilst we considered the number of individuals that had eclosed and their mass for each week in our analyses, for dead individuals we only considered the total dead by the end of the experiment for two reasons: (i) if an individual dies underneath the brood or in an inconspicuous place then the date of death is difficult to accurately determine; and (ii) dead individuals decompose resulting in inaccurate measures of mass.

### Statistical Analyses

For all analyses diet was considered as the predictor. Error structures were adjusted according to the response variables, with linear mixed effects models (LMER) using a gaussian error distribution for continuous response variables (consumption, individual mass, and colony mass) and generalized linear mixed effects models (GLMER) with a Poisson distribution (link function = “log”) for count variables (numbers of adult individuals and pupae). To analyze total worker mortality by the end of the experiment, we used a GLMER with binomial error distribution. Experimental replicates (*block*) were incorporated as a fixed factor in the models. For analysis of repeated weekly measures (colony mass, weekly consumption, weekly worker eclosion, and weekly worker mass) we: (i) included observation *week* nested in *colony* as a random factor to account for temporal pseudoreplication of colony measures; and (ii) ran each as a linear and second order polynomial (quadratic) model, choosing the best fitting model based on AIC values. When considering mass of individuals, male wet mass was log10 transformed in response to non-normal distribution of the data. For models of worker production over time and end-point total numbers between diets, we compared models with and without colony starting pupae number as an explanatory covariate. Statistical outputs stated in the main text are from models that did not include starting pupal number, as outputs showed the same significance patterns (Supplementary Tables 9, 10). All analyses were conducted in R v3.5.1 (55) with packages “lme4” v1.1.21 for mixed effects models (56), “lmerTest” v3.1.0 to calculate corresponding *p*-values (57) and “ggplot2” for data visualization (58).

## Results

### Pollen Analysis

We identified a total of 16 pollen morphotypes in the supplied honeybee collected pollen (Supplementary Figure 1), with ER1 and ER2 polyfloral diets consisting of 14 and 13 morphotypes, respectively. Nine of the 14 morphotypes (ER1) constituted 2.2–37.8% composition of the pollen diet based on relative pollen grain counts [Shannon-Weiner Index (H) of pollen community = 1.90]. Eleven of the 13 morphotypes (ER2) constituted 2.2–19.6% (H = 2.23). ER1 monofloral diet consisted of five of the total 16 morphotypes with one of these morphotypes (morph A) constituting 96.8% of the diet (H = 0.17). For the ER2 monofloral diet, a single morphotype (morph F) constituted 100% of the diet (Supplementary Table 5). The Bradford assay, based on the mean crude protein content of the pollen lysate (mg/mL), showed the monofloral diets had a 1.8 and 1.5-fold higher relative protein concentration than the polyfloral for diets compared in ER1 and ER2, respectively (mean value: ER1 = 59.4 vs. 32.9 mg/mL; ER2 = 63.2 vs. 41.5mg/mL; Supplementary Table 6).

### Food Consumption

**Figure 1:**
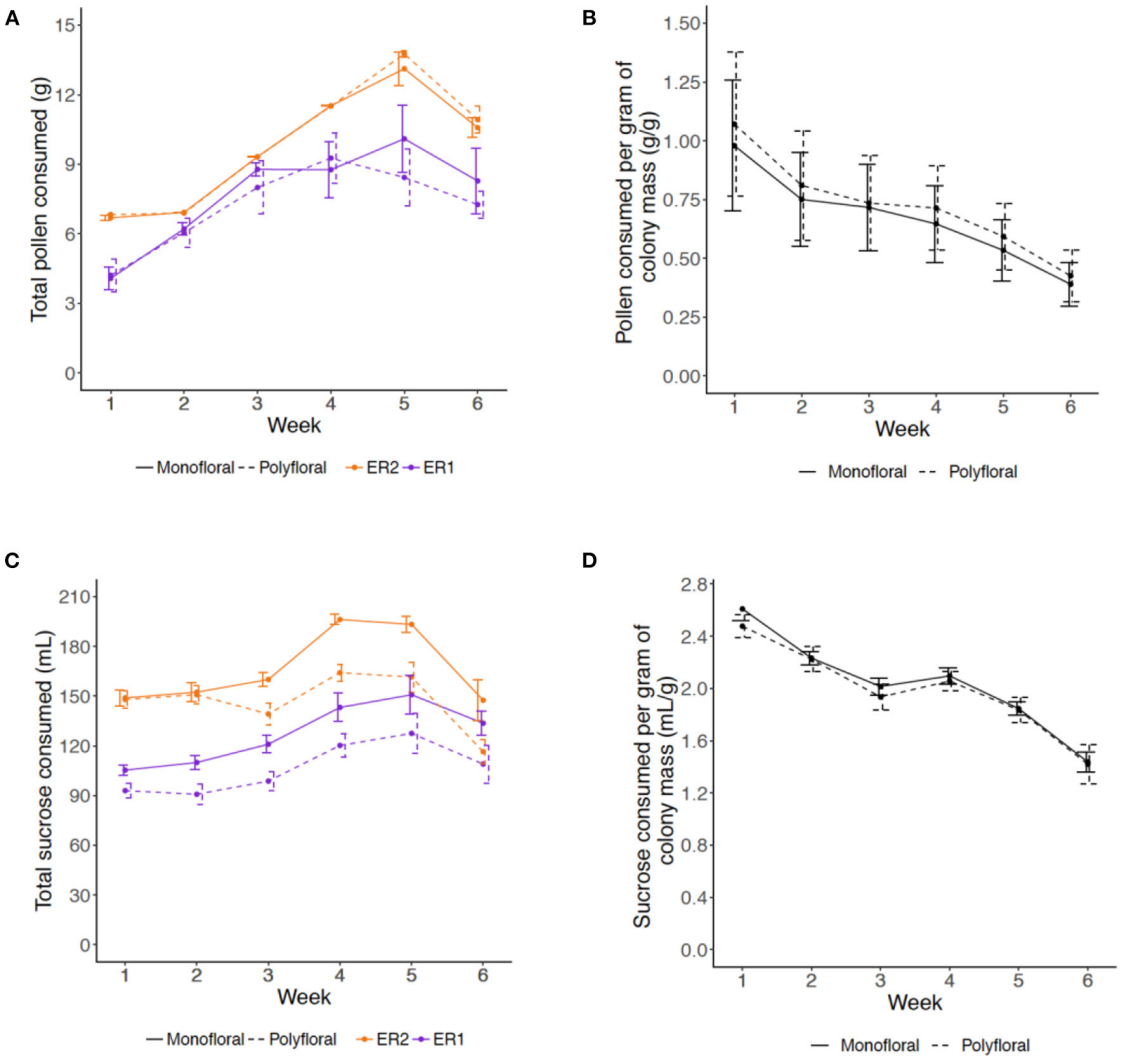
Weekly amounts of **(A,B)** pollen and **(C,D)** 40% sucrose solution consumed by colonies over the course of the 6-week experiment. **(A)** Mean (±s.e.m) of the total mass (grams) of provisioned pollen consumed per colony per week (monofloral = solid line; polyfloral = dashed line; ER1 = purple; ER2 = orange); **(B)** Relative amounts of pollen consumed per unit of colony mass (gram per gram) for monofloral and polyfloral colonies (monofloral = solid line; polyfloral = dashed line); **(C)** Mean (±s.e.m) of the total volume (mL) of 40% sucrose solution consumed per colony per week (monofloral = solid line; polyfloral = dashed line; ER1 = purple, ER2 = orange); **(D)** Relative volume of 40% sucrose solution consumed per unit of colony mass (mL per gram) for monofloral and polyfloral colonies (monofloral = solid line; polyfloral = dashed line).

Consumption of pollen and sucrose solution showed a parabolic type trend, increasing over the first 4–5 weeks of the experiment and decreasing in weeks 5 and 6 (LMER: *week*^2^: pollen, t = −5.56, *p* < 0.001; sucrose, t = −4.54, *p* < 0.001). For pollen consumption, there was no significant difference between monofloral and polyfloral colonies (*treatment*^2^: t = 0.29, *p* = 0.77) and this was consistent over the duration of the experiment (treatment^*^week^2^: t = 0.04, *p* = 0.97; Figure 1A, Supplementary Table 7), with colonies consuming a median (IQR) of 95.1% (77.0–97.5) vs. 88.5% (76.6–99.0) of the total pollen provided, respectively. For sucrose consumption, monofloral consumed significantly more than polyfloral colonies (*treatment*^2^: t = 3.92, *p* = 0.001), which remained relatively consistent over the course of the experiment (treatment^*^week^2^: t = 0.96, *p* = 0.34; Supplementary Figure 1B, Supplementary Table 7), with 88.1% (82.4–94.5) vs. 76.6% (69.7–82.3) of the total provisioned volume of sucrose consumed.

We then analyzed relative consumption by taking the amount consumed per gram of colony mass (total mass (pollen) or volume (sucrose) divided by colony mass each week). Relative pollen and sucrose consumption significantly decreased over the consecutive weeks (LMER: *week:* t = −2.79, *p* = 0.011 and t = −8.21, *p* < 0.001, respectively), but this response did not significantly differ between monofloral and polyfloral colonies (*treatment*: t = 0.18, *p* = 0.86 and t = −0.75, *p* = 0.46) and did not change over time (treatment^*^week: t = −0.13, *p* = 0.90 and t = −0.51, *p* = 0.62; Figures 1C,D, Supplementary Table 7).

### Colony Growth

**Figure 2:**
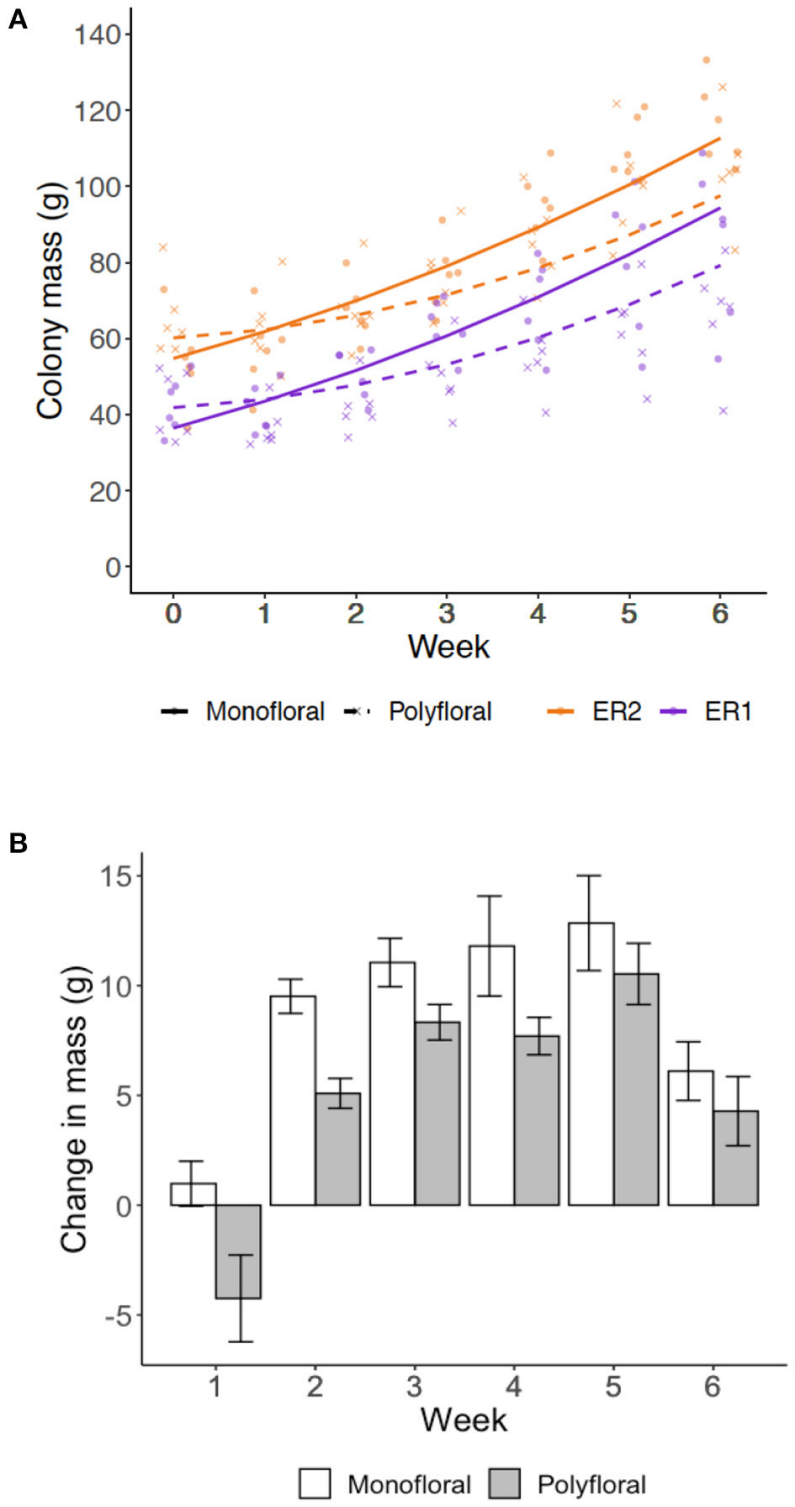
Colony growth rates. Weekly measures of colony mass (grams) for the colonies provisioned a monofloral (*n* = 12) and polyfloral (*n* = 12) diet. **(A)** Scatter plot of cumulative colony growth rate with fitted lines representing the LMER estimates (monofloral = solid line/dot symbol; polyfloral = dashed line/cross symbol; ER1 = purple lines/symbols; ER2 = orange lines/symbols); **(B)** Mean (±s.e.m.) colony weight change (grams) by the end of each week based on raw colony mass measures.

**Figure 3:**
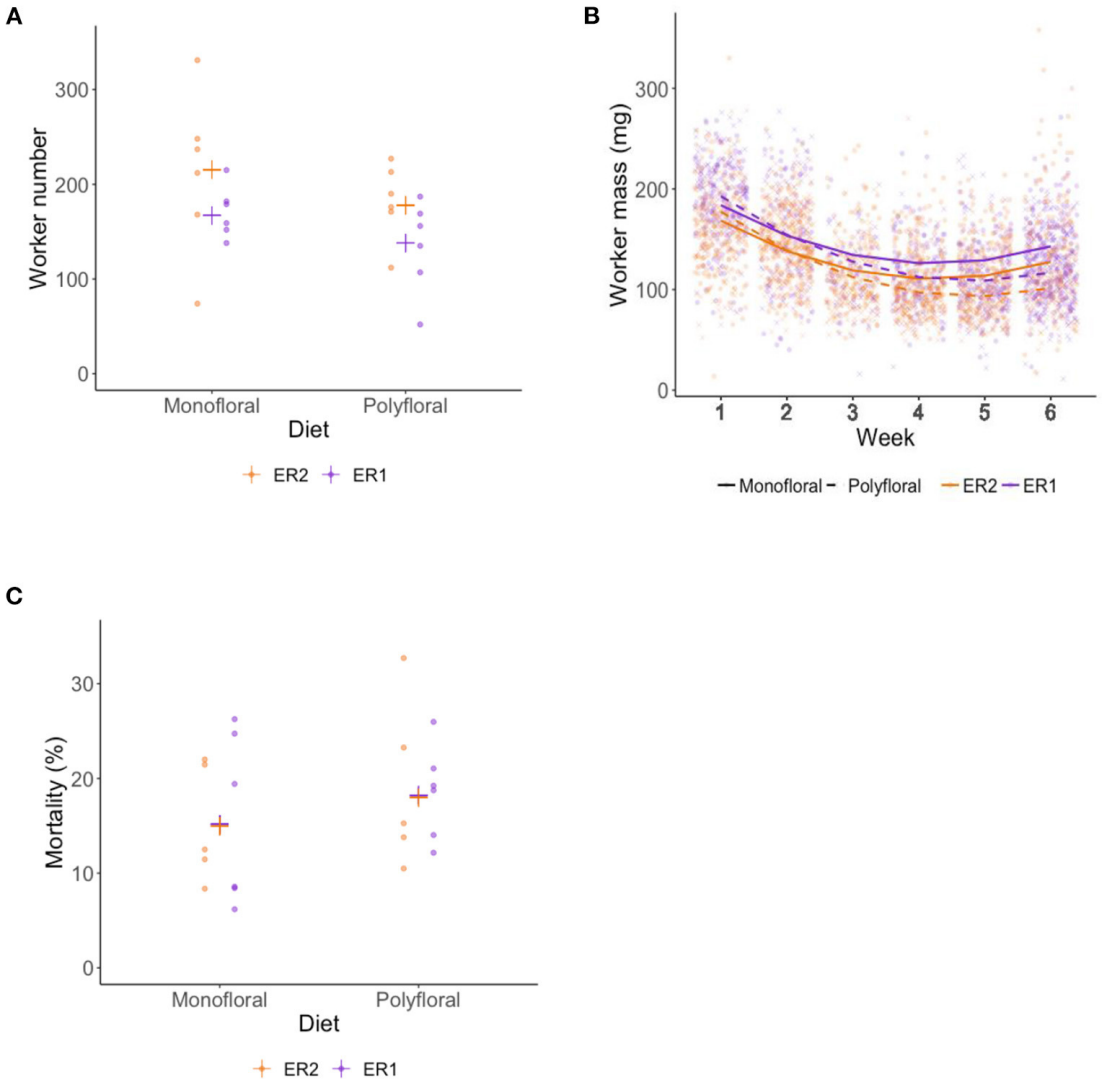
End of experiment comparisons of **(A)** total adult workers reared (worker production), **(B)** wet mass (milligrams) of workers, and **(C)** percentage mortality of total adult workers at end of the 6-week experiment. **(A)** Raw data points for the total number of adult workers reared by each monofloral (*n* = 12) and polyfloral (*n* = 12) colony (ER1 = purple symbols, ER2 = orange symbols), with the estimated mean (cross symbols) from back transformation of the GLMER; **(B)** Scatter plot showing mass of workers that eclosed in each week of the experiment. Fitted lines represent the LMER estimates (monofloral = solid line; polyfloral = dashed line), and the data points per week are jittered (monofloral = dot symbol; polyfloral = cross symbol; ER1 = purple lines/symbols; ER2 = orange lines/symbols); **(C)** Raw data points for percentage mortality of total adult workers for each monofloral (*n* = 11) and polyfloral (*n* = 11) colony (ER1 = purple symbols, ER2 = orange symbols), with the estimated mean (cross symbols) from back transformation of the GLMER. Note that one monofloral and one polyfloral colony were removed from analysis due to a mortality rate of >50%.

Over the course of the experiment our model suggests that the weekly colony growth rate (positive linear term) was significantly higher in monofloral relative to polyfloral colonies (LMER: treatment^*^week: t = −3.23, *p* = 0.004; Figure 2A, Supplementary Table 8). Accordingly, mean polyfloral colony mass was 18.84 g (−22.1%) and 11.34 g (−9.8%) lower relative to monofloral colonies in ER1 and ER2, respectively (Figure 2B). Cumulative numbers of weekly worker eclosure did not differ significantly between monofloral and polyfloral colonies (GLMER: *treatment*: t = 1.64, *p* = 0.10; Supplementary Table 9). However, there were consistent negative model estimates for polyfloral colonies, and when comparing endpoint data (cumulative total of workers produced over the whole experiment), we found monofloral colonies had reared a significantly higher number of workers compared with polyfloral colonies (GLMER: *z* = −6.17, *p* < 0.001; Figure 3A); an effect found when also considering colony starting pupae number (*z* = −4.22, *p* < 0.001; Supplementary Table 10).

Worker mass was taken from all workers alive at the end of the experiment for which their tag could be successfully identified (*n* = 3,356). Overall, mean worker mass showed a decrease over the first 3 weeks (likely due to pollen provision being limited) but appeared to plateau over the latter 3 weeks of the experiment (LMER: *week*^2^: t = 16.35; *p* < 0.001; Figure 3B). Our model suggests that the rate of decreasing mass (linear term) was significantly greater in polyfloral relative to monofloral colonies (treatment^*^week: t = −2.70, *p* = 0.013; Supplementary Table 11). Indeed, when pooling all workers that eclosed in weeks 1–3, worker mass between monofloral and polyfloral colonies was similar with a mean difference of 0.06% (mean ± s.e.m.: 150.1 ± 1.6 vs. 149.9 ± 1.8 mg), but workers that eclosed during weeks 4–6 in polyfloral colonies had a 17.9% lower mass compared with workers eclosed in monofloral colonies during that time period (124.5 ± 1.2 vs. 102.3 ± 1.2 mg).

Analysis of total worker mortality during the 6-week experiment revealed lower mortality in monofloral relative to polyfloral colonies (GLMER: treatment: *z* = −2.79, *p* < 0.01; Figure 3C, Supplementary Table 12). Note that the mortality analysis shown here considered 22 of the 24 colonies, as one monofloral and one polyfloral colony had mortality rates of 55 and 52%, respectively, and were not considered (due to not meeting model assumptions). However, analysis considering all 24 colonies showed the same significant trend (Supplementary Table 12).

**Figure 4:**
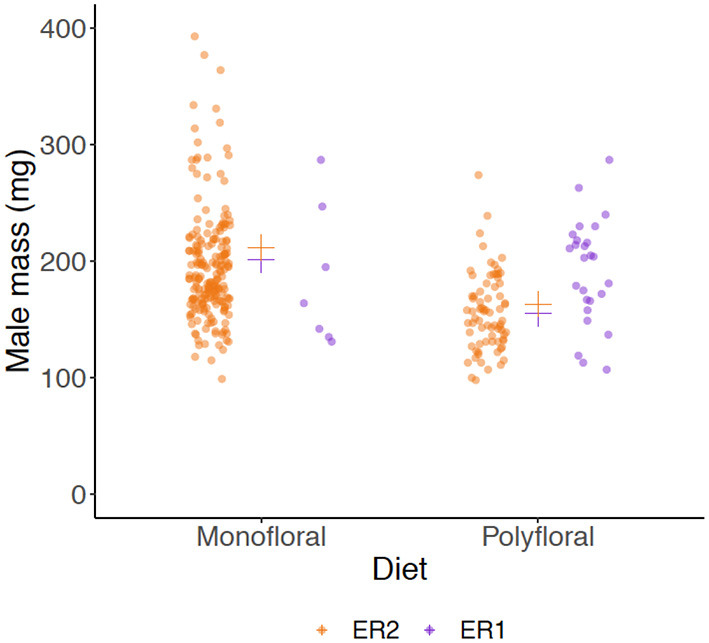
Wet mass (milligrams) of males (drones). Scatter plot showing mass of all adult males that had been produced by the end of the experiment between monofloral (*n* = 10) and polyfloral (*n* = 7) colonies. Jittered raw data points are shown (ER1 = purple symbols; ER2 = orange symbols), with the estimated mean from back transformation of the LMER overlaid (cross symbols).

Males eclosed in 10 monofloral and seven polyfloral colonies (Fishers Exact Test: *p* = 0.37). The median (range) number of males produced by colonies was higher in monofloral compared with polyfloral colonies [4 (1–108) vs. 1 (1–47); Supplementary Table 13], but the high ranges showed a heavy right skew in the data with a few colonies producing a particularly high number of males, making interpretation of this effect difficult. Interestingly, in parallel to our finding of lower worker mass in polyfloral colonies, we found a trend toward males of lower mass being reared by polyfloral relative to monofloral colonies (*n* = 308, LMER: *treatment*: t = −2.10, *p* = 0.063; Figure 4, Supplementary Table 14). No gynes were recorded to have eclosed in any colony.

**Figure 5:**
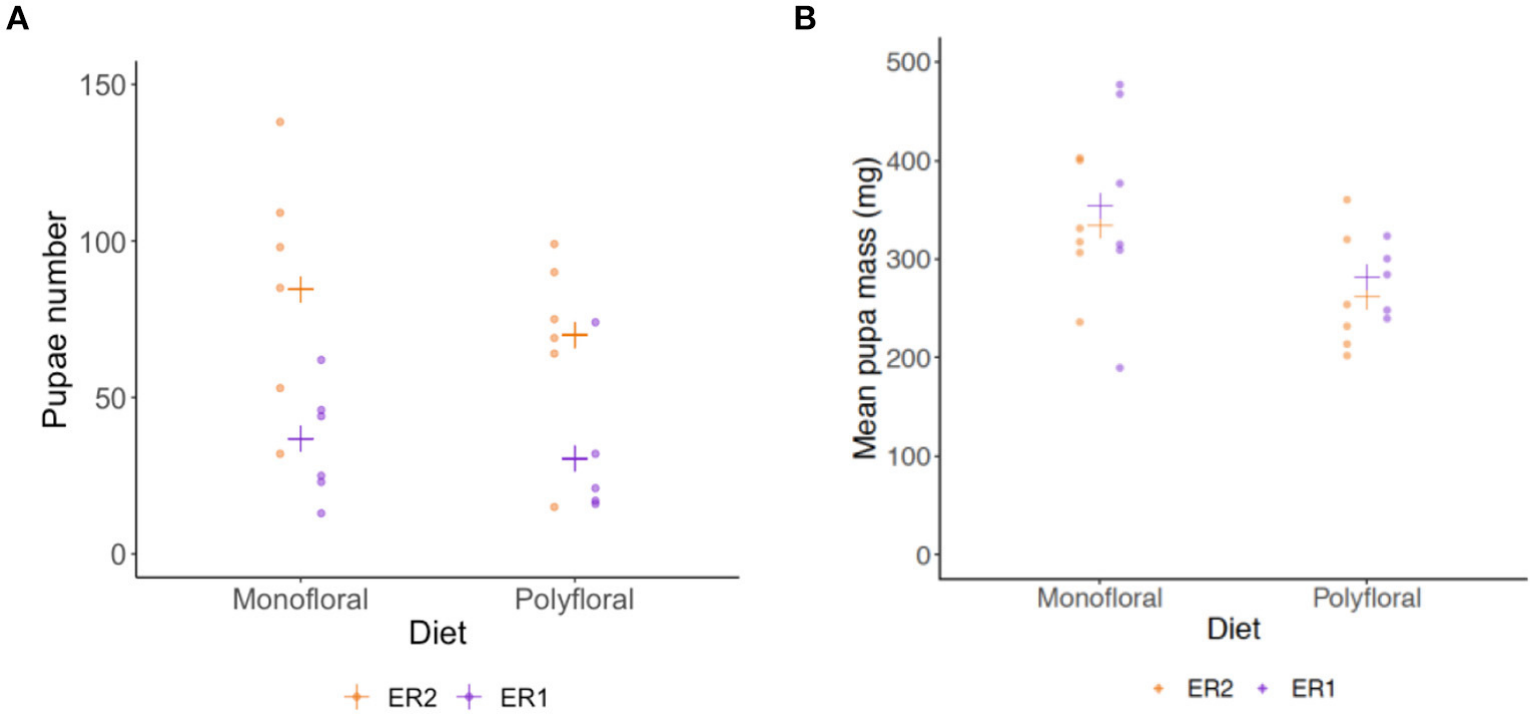
End of experiment comparisons of **(A)** total number of pupae, and **(B)** mean wet mass (milligrams) of pupae in monofloral (*n* = 12) and polyfloral (*n* = 11) colonies. Raw data points are shown (ER1 = purple symbols; ER2 = orange symbols), with estimated mean (cross symbols) from back transformation of the GLMER (N.B. We removed one polyfloral colony that had produced gyne pupae).

Dissection of colonies at the end of the experiment revealed monofloral colonies possessed a significantly higher number of pupae relative to polyfloral colonies (GLMER: treatment: *z* = 3.39, *p* < 0.001; Figure 5A, Supplementary Tables 10, 14). These pupae also had a significantly higher mean pupal wet mass (LMER: *treatment*: t = −2.41, *p* = 0.026; Figure 5B, Supplementary Table 15). Note that one polyfloral colony did possess one new gyne pupae (~600 mg), which was excluded from the above analysis.

## Discussion

Our experiment simulated a hypothetical scenario whereby bee colonies had access to a set quantity of pollen resource with half the colonies having access to pollen from multiple floral species (polyfloral) and the remaining half restricted to pollen from a single species but with a 1.5–1.8 times higher relative crude protein concentration (monofloral). Over the 6 weeks we found that for nearly every colony growth measure, monofloral performed significantly better than polyfloral colonies. This included higher worker mass and lower mortality, which suggests that in the context of colony growth dynamics in a controlled low-challenging environment, crude protein concentration of pollen can potentially outweigh access to a higher diversity of florally sourced pollen.

### Colony Development, Mortality and Male Production

We found a higher cumulative growth rate in respect to colony mass in monofloral colonies, which was also reflected by a higher number of workers being reared by the end of the experiment. Generally, we found no clear evidence that low diversity of florally sourced pollen (monofloral) had a negative effect on our colony measures over a 6-week period (although colony mass between monofloral and polyfloral colonies appeared to show a more paralleled rate of growth in the latter couple of weeks). If diversity was beneficial in the longer-term, we might have expected to see pupae number and mean pupae mass to have been similar, if not higher, in polyfloral compared to monofloral colonies by the end of the experiment. Instead, we found polyfloral colonies to have significantly lower pupal number and mass at the end of the experiment. Considering previous findings by Dance et al. (49) showing increased brood numbers in queenless *B. terrestris* microcolonies fed a polyfloral and not monofloral diet (although of similar crude protein content), our findings indicate that whilst bumblebee colony growth may be able to cope with reduced pollen diversity it is dependent on the “right” floral species being available (24, 32, 33, 36, 37).

Despite our controlled lab study imposing no intentional stress on workers, we found worker mortality to be higher in polyfloral colonies. This finding was surprising given pollen diversity has previously been shown to improve worker condition and increase longevity in honeybees (*Apis mellifera*) (44, 45), and considering we were able to distinguish 16 pollen morphotypes in our polyfloral diets we presume nutrient diversity will have been high [e.g., amino acids, lipids and sterols (41, 42, 59)]. A possibility is that our monofloral sources of pollen could each represent a kind of “superfood” containing all or many of the essential nutrients required for bumblebee health. Interestingly, previous studies have found large variation for individual and micro-colony level responses to pollen sourced from different plant species (24, 32–38). Alternatively, the difference observed in worker mortality may have been caused by differences in protein availability between the lower protein content in the polyfloral diet and higher protein content in the monofloral. Given that bumblebee workers have a dietary requirement for protein (60), and that bees fed on our polyfloral diet had overall relatively less access to protein, it is possible that workers on our polyfloral diet were unable to meet their required protein intake, resulting in a higher mortality. We should also consider that whilst mortality was lower in monofloral colonies in our controlled laboratory setup, differences may not manifest or diet effects may switch when under more challenging conditions, with workers from polyfloral colonies possibly being more resilient to stress, such as exposure to pathogens or pesticides (41, 49).

The production of sexuals underpins colony fitness and changes in diet are likely to be a key determining factor, and our counts of male production per colony could be considered as a fitness proxy (61). A previous study by Dance et al. (49) reported a monofloral diet to have a negative effect on males (although crude protein was similar with comparative polyfloral diet: 10.2 vs. 12.6%) with colonies rearing fewer and lighter individuals. In contrast to this, we found no difference in the number of males reared between monofloral and polyfloral colonies, and in fact male body mass was actually higher for those reared in monofloral colonies. Considering smaller males can have a lower probability of mating (61–63), our findings reiterate the point that access to the right floral species, rather than floral diversity, may be vital for determining colony fitness (64). If the experiment had run longer, we may have observed gyne (new queen) production. Given the higher mass of males observed in colonies fed on the high protein monofloral diet, we predict this diet may also have supported rearing of larger gynes, or the decision to rear gynes at all. That said, if key nutrients are missing from the monofloral diet, there may be the potential to affect the ability of queens to hibernate or establish a nest the next year (65, 66). If revealed, this could suggest longer-term benefits of diet diversity over those observed in this study.

### Individual Number: Size Trade-Offs

Regarding worker production, Herrmann et al. (50) showed that mean mass of workers is important for the later production of gynes in bumblebee colonies (*Bombus impatiens*). We may therefore expect that under low food resources, such as reduced amount of absolute crude protein income, a colony may decide to alter the number of individuals it rears in order to maintain a certain average mass of reared individuals. However, we found that polyfloral colonies, that had access to a lower amount of crude protein, reared both a lower number and mass of workers—particularly in the latter 2 weeks of our experiment (a similar pattern was observed for reared males). Producing a smaller and lighter workforce in polyfloral colonies may have implications for colony task performance, given that larger workers appear to show better learning and foraging performances [(67–72); but see (73)]. The lack of any apparent compensatory strategy observed in our study is unlikely to be down to a reduced appetite, as the per capita consumption rate of both sucrose solution and pollen was not significantly different and actually higher on average in polyfloral colonies (Figures 1B,D). Perhaps, therefore, the decrease in worker number and/or worker mass was not of concern to the colony with mean worker mass large enough to still contribute to the primary tasks required for colony functioning. Or, since workers could not leave the confines of our lab reared colonies, maybe there was a lack of stimulus to implement such a trade-off, given total mass of pollen income to the colony was consistent. On the other hand, the protein level in polyfloral colonies could have been low enough that any trade-off with mass would have lowered worker numbers to a point of having a substantial detrimental effect to colony function. To more formally test this, a gradient of crude protein concentrations would be needed.

### Future Work and Implications of Our Findings

Investigating the dynamics of full-sized queenright colonies is practically and financially challenging, but future work will benefit from increasing treatments and implementing a set of crossed-designed experiments to: (i) undertake a combination of high and low protein concentrations with high and low pollen diversity to better identify the threshold trade-offs between pollen diversity and protein concentration; and (ii) investigate how variation among the other pollen nutrients may compensate for a reduced diversity of florally sourced pollen [i.e., key amino acids, nitrogen content, lipids (fatty acids) and sterols (24, 41, 42, 59)]. When considering point ii, it was interesting to find that the two different monofloral pollen species showed a consistent positive effect (with similar effect size) on colony development compared to polyfloral colonies, despite the likelihood of some aspect of nutrient composition differing between them. This reinforces the importance of high crude protein concentration for colony development. However, further studies are needed to assess the response of individuals to manipulated stress when reared under diets similar to ours. In doing so, it will improve our understanding of the relative importance of pollen diversity vs. protein concentration under varied challenging scenarios (36, 41). Different pollen species can, however, have different effects on bee health [e.g., (51)]. Given that our polyfloral diets contained up to 16 morphotypes, a worthy research avenue is to determine how each individual pollen species may affect bumblebee colonies. The pollen isolated to make up the monofloral pollen diets constituted a relatively high proportion of the pollen that was ordered from the commercial supplier, and is a composition representative of that collected by honeybee foragers from commercial hives. Considering the benefit(s) reported in our study, it is highly plausible that honeybee foragers had a preference to visit this floral species or genus in their surrounding landscape, whether because it was in high abundance and/or they were attracted to it over others in the area (74). Given that honeybee and bumblebee foraging niches often overlap (75, 76), bumblebees are likely to visit flower species contained in our honeybee collected pollen, and thus bumblebees encountering the species we used in our monofloral diets is highly probable. We note that the color and grain morphology of the purple pollen (ER1 monofloral) looks similar to pollen sourced from plants in the *Phacelia* genus, which are commonly grown in agricultural areas across Europe as either a cover crop and/or a constituent of flowering field margins (77–79). If our deduction is correct, we can tentatively suggest that high coverage of *Phacelia* plantings over a long enough time span and appropriate phenology could be a good food source to support colony growth of bumblebees. Indeed, bumblebee species have been shown to be able to detect and preferentially forage on floral species that provide high protein content and essential nutrients they require if available (80–84).

In conclusion, when designing insect pollinator and particularly bumblebee suitable landscapes, our study supports the view that we cannot necessarily assume higher floral diversity is always better in terms of supporting specific pollen dependent insects, unless we consider the nutritional content of each of the composite floral species (22, 24).

## Data Availability Statement

The original contributions presented in the study are included in the article/Supplementary Materials. Regarding data analyses, the R scripts and associated data files are available on FigShare (10.6084/m9.figshare.c.5698819).

## Author Contributions

CW, AR, JC, and RG conducted the experiments. CW, AR, AA, and RG carried out the pollen and data analyses. CW and RG wrote the paper. All authors contributed to the article and approved the submitted version.

## Funding

This study was supported by a Royal Society research grant (RG130455) awarded to RG. AA and AR were supported by a NERC new investigator grant (NE/L00755X/1) awarded to RG.

## Conflict of Interest

The authors declare that the research was conducted in the absence of any commercial or financial relationships that could be construed as a potential conflict of interest.

## Publisher's Note

All claims expressed in this article are solely those of the authors and do not necessarily represent those of their affiliated organizations, or those of the publisher, the editors and the reviewers. Any product that may be evaluated in this article, or claim that may be made by its manufacturer, is not guaranteed or endorsed by the publisher.
