# Supplementary material for: Pollen Source Richness May Be a Poor Predictor of Bumblebee (Bombus terrestris) Colony Growth
Source: Front Insect Sci. 2021 Dec 1;1:741349. doi: 10.3389/finsc.2021.741349 (PMC10926443; doi:10.3389/finsc.2021.741349)
Supplement: Supplementary file 1 [file Data_Sheet_1.pdf]

Pollen source richness may be a poor predictor of bumblebee (*Bombus terrestris*) colony growth

Watrobska, Ramos Rodrigues, Arce, Clarke & Gill

Supplementary Methods

Bradford assay for determining protein concentrations

We investigated what the relative differences were in crude protein content between the pollen diets by conducting a Bradford protein assay (Bradford 1976). We first prepared the samples for analysis by washing 1g of each pollen diet in 10mL of ultrapure Milli-Q water and vortexed the samples until the pellets had dissipated and the pollen grains had entered suspension. The suspension was centrifuged at 6000rpm for 5mins and the supernatant was discarded. The remaining pollen was left to dry overnight at room temperature (25°C) under a fume hood. We then placed between 97-100mg of pollen into individual 2mL homogenisation tubes, containing 600mg of 1mm ceramic homogenisation beads. The tubes were placed at -80°C for 40mins prior to homogenisation at 30MHz for 5mins (Qiagen Tissue Lyser II). After the initial homogenisation, we added 0.1M NaOH to create approximately 1mL of a 0.1mg/mL pollen lysate which was heated to 100ºC for 5mins before re-homogenising the lysate for a further 3mins. We then conducted the Bradford assay using the Bio-Rad Protein Assay Kit microassay 300µL microplate protocol using bovine serum albumen as the protein standard (Bio-Rad Laboratories). Because of the high protein concentration of the pollen, we conducted the assay on the lysate after serial dilution in Mili-Q water by a factor of 10^{-3}. We assayed each pollen sample in triplicate wells containing 40µL Bradford reagent mixed with 160µL of pre-prepared BSA protein standards (0-80µL/mL). The plates were incubated at room temperature for 5mins, followed by 30secs of shaking, before reading the absorbance at 595nm (spectrophotometer,

Synergy HT, Bioteck). Protein concentrations were calculated using linear regression analysis from the protein standards.

Preparation of pollen samples for microscopy

The following protocol was undertaken to prepare samples for observation under the microscope: a 2g subsample of each pollen diet (ER1 monofloral, ER1 polyfloral, ER2 monofloral, ER2 polyfloral) was taken and placed in a separate 50mL falcon tube along with 20mL of deionised water. Each sample was then vortexed for 1min to produce a well-mixed homogenous 'pollen stock solution' (at a 0.1mg/µL concentration). A 'fuchsine and spore solution' was previously prepared: with a 25mg/mL 'spore suspension stock' first being prepared by dissolving *Lycopodium* spores in deionised water; followed by adding a volume of 'fuchsine and Ethanol stain solution' (0.01g/mL) to obtain a final fuchsine and spore solution at a concentration of 1.25mg/mL. Note: the highly consistent diameter size of *Lycopodium* spores (33µm) were integrated into the sample to provide a standardised size marker, and the fuchsine dye was used to increase contrast and so enhance identification of different pollen grain features (Sawyer and Pickard 2006). From each pollen stock solution 10µL was aliquoted into separate wells of a multi-well microplate with an additional 90µL of the 'fuchsine and spore solution' added to each. This 100µL sample was left to stain for 5mins. For each diet, 3µL of the 100µL sample was pipetted out on to a 75x25mm glass microscope slide to provide a sample spot which was repeated four times. Once the sample spots were dry, 10µL of warmed 80% glycerol was pipetted on top of the sample and an 18x18mm cover slip was placed over the top.

References

Bradford MM (1976) A rapid and sensitive method for the quantitation of microgram quantities of protein utilizing the principle of protein-dye binding. Anal Biochem 72:248–254. https://doi.org/10.1016/0003-2697(76)90527-3

Sawyer R, Pickard RS (2006) Pollen identification for beekeepers. Northern Bee Books, Mytholmroyd, West Yorkshire.

Supplementary Figures

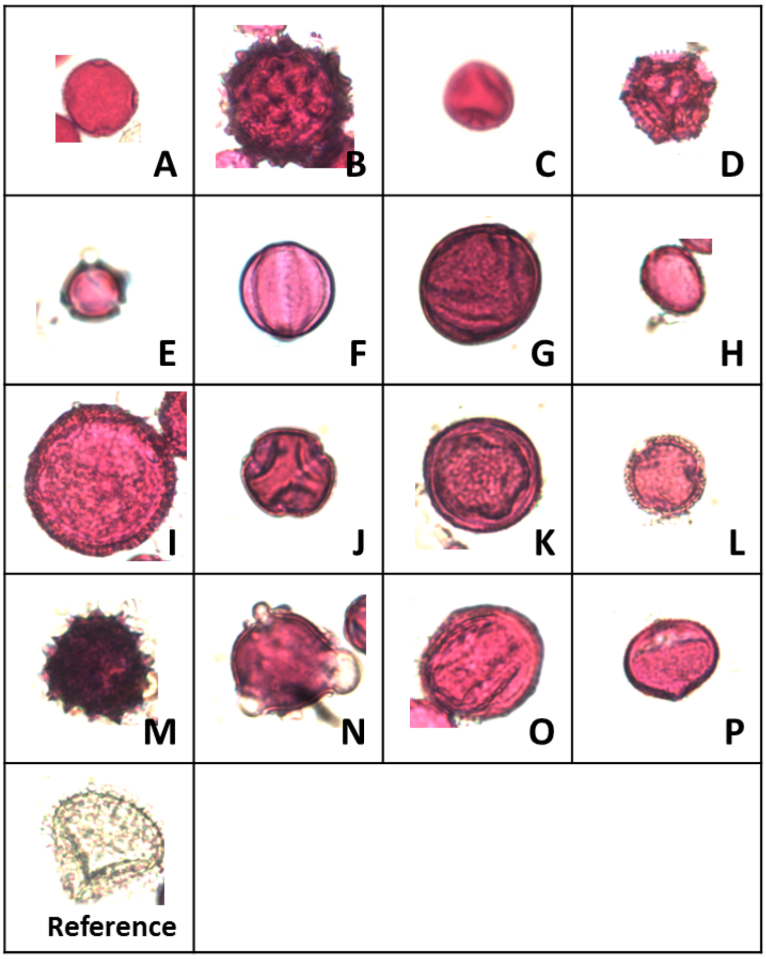

Figure S1. The 16 different pollen grain morphotypes identified in the honeybee collected pollen used across all four pollen diets in this experiment. Pollen was stained using a fuchsine dye and observed at ×400 magnification, with each image cropped (and hence not to scale) to show the pollen grain morphotype of interest. The reference panel shows a *Lycopodium* spores which was used as a size reference as it consistently measures 33µm at the widest point. Please see Table S5 for pollen grain diameters. Morphotype A was the dominating species in the ER1 monofloral pollen diet, and morphotype F was the dominant in ER2 monofloral diet.

Supplementary Tables

Table S1. **Census of each colony on arrival.** ER2 colonies arrived larger than expected, and so eleven of the twelve colonies were culled (by random removal of workers) to the size of the twelfth smallest colony.

Experimental replicate	Colony identification	Diet	Initial colony mass (g)	Estimated pupal cell number	Arrival worker number	Worker number post-cull
1	A	Polyfloral	51.06	31	27	N/A
1	B	Polyfloral	35.76	15	25	N/A
1	C	Polyfloral	36.96	29	24	N/A
1	D	Polyfloral	32.81	27	20	N/A
1	E	Polyfloral	49.26	35	21	N/A
1	F	Polyfloral	52.14	24	20	N/A
1	G	Monofloral	52.73	44	25	N/A
1	H	Monofloral	47.56	41	26	N/A
1	I	Monofloral	39.21	42	25	N/A
1	J	Monofloral	37.34	31	20	N/A
1	K	Monofloral	46.08	21	33	N/A
1	L	Monofloral	33.16	26	19	N/A
2	AA	Monofloral	57.10	66	60	35
2	BB	Monofloral	72.98	52	48	35
2	CC	Monofloral	55.14	31	62	35
2	DD	Monofloral	52.47	35	41	35
2	EE	Monofloral	50.83	36	36	35
2	FF	Monofloral	36.56	33	35	N/A
2	GG	Polyfloral	67.50	44	51	35
2	HH	Polyfloral	83.91	73	46	35
2	II	Polyfloral	57.18	34	52	35
2	JJ	Polyfloral	57.52	21	57	35
2	KK	Polyfloral	62.75	25	49	35
2	LL	Polyfloral	61.55	26	48	35

Table S2. Statistical outputs from linear and generalised linear models (GLM & LM) in R when analysing the difference in the a) number of workers after the cull, b) estimated number of pupal cases and c) colony mass between colonies assigned to a monofloral (intercept) and polyfloral diet on arrival. Asterisks highlight significant differences (alpha values: 0.05 * 0.01 ** 0.001 ***). ER = Experimental Replicate

a) GLM (worker number ~ diet + ER, family = Poisson)					
	**Estimate**	**S.E.**	**z value**	**P value**	
(Intercept)	3.875	0.054	72.139	<2e-16	***
dietpoly	0.023	0.068	0.339	0.735	
ER1	-0.718	0.072	-9.955	<2e-16	***

b) GLM (pupae number ~ diet + ER, family = Poisson)					
	**Estimate**	**S.E.**	**z value**	**P value**	
(Intercept)	3.765	0.056	67.654	<2e-16	***
dietpoly	-0.176	0.069	-2.547	0.011	*
ER1	-0.263	0.070	-3.780	0.000	***

c) LM (colony mass ~ diet + ER)					
	**Estimate**	**S.E.**	**t value**	**P value**	
Intercept	56.864	3.467	16.402	1.9e-13	***
dietpoly	5.520	4.003	1.379	0.182	
ER1	-16.868	4.003	-4.214	0.000	***

Table S3. Mass of pollen (g) and volume of 40% sucrose solution provisioned to each colony across both experimental replicates.

	ER1		ER2	
**Experimental day**	**Pollen (g)**	**Sucrose (mL)**	**Pollen (g)**	**Sucrose (mL)**
1	2.0	40	3.0	60
4	1.6	36	2.0	50
6	2.0	40	2.0	50
8	3.0	54	3.0	75
11	2.0	40	2.0	50
13	2.0	40	2.0	50
15	4.0	60	4.0	75
18	2.7	40	2.7	50
20	2.7	40	2.7	50
22	5.0	70	5.0	90
25	3.3	47	3.3	60
27	3.3	47	3.3	60
29	6.0	80	6.0	90
32	4.0	53	4.0	60
34	4.0	53	4.0	60
36	7.0	90	7.0	105
39	4.7	60	4.7	70
42	Colonies sacrificed			

Table S4. A) Descriptions of the 16 pollen morphotypes found across all four diets. Morphotype features are described based on categories outlined in Sawyer (2006): size category (very small <20µm, small 20-30µm, medium 30-50µm, large 50-100µm, very large >100µm), shape, thickness and structure of the exine (outer layer) and number of apertures. Level of staining relates to the level at which the morphotype absorbs the fuchsine dye. **B)** Key used to determine the level of staining of a pollen grain with fuchsine dye, with example morphotypes.

A)

Feature	A	B	C	D	E	F	G	H
**Size (µm)**	19.78	42.74	19.01	26.83	13.75	20.16	38.98	19.66
**Size category (µm)**	Very small <20	Medium 30-50	Very small <20	Small 20-30	Very small <20	Small 20-30	Medium 30-50	Very small <20
**Shape**	Round	Irregularly round	Round	Hexagonal	Tri-lobed	Round	Oval flattened	Rounded triangular
**Thickness and structure of exine**	Thin	Broad-based spines	Thin	Small projections	Medium, no rods	Thin	Medium, no rods	Medium, no rods
**Number of apertures**	4-6	Indefinite	0	Indefinite	3	0	0	0
**Level of staining**	2	3	2	3	1	1	4	1

Feature	I	J	K	L	M	N	O	P
**Size (µm)**	48.38	18.28	38.02	26.28	29.78	30.00	35.07	27.08
**Size category (µm)**	Medium 30-50	Very small <20	Medium 30-50	Small 20-30	Small 20-30	Small 20-30	Medium 30-50	Small 20-30
**Shape**	Round	Irregularly round	Round	Round	Irregularly round	Tri-lobed	Oval elongated	Irregular
**Thickness and structure of exine**	Spaced rods	Medium, no rods	Thin	Beaded	Broad-based spines	Medium, no rods	Medium, no rods	Medium, no rods
**Number of apertures**	0	3	0	0	Indefinite	3	0	0
**Level of staining**	2	3	3	1	4	3	3	3

B)

Level of staining	Description	Example
1	Light	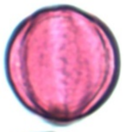 Morphotype F
2	Medium	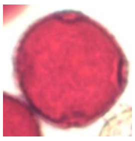 Morphotype A
3	Medium with some darker patches	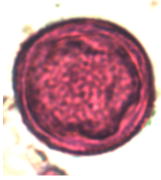 Morphotype K
4	Dark	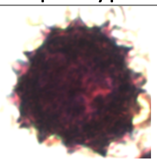 Morphotype M

Table S5. Composition of each diet by morphotype. Size (µm) was calculated by measuring a single pollen grain of each morphotype relative to a *Lycopodium* spore (33µm at widest point) using the software ImageJ (NIH). The table shows the raw count of pollen grains per morphotype (count), and the percentage compositions wen based on the raw count (%) and when based on the count weighted by grain size (relative %).

Morph.	Size	Polyfloral pollen diet						Monofloral pollen diet					
		ER1			ER2			ER1			ER2		
		count	%	relative %	count	%	relative %	count	%	relative %	count	%	relative %
A	19.8	37	13.6	9.1	1	0.7	0.5	512	97.9	96.8	-	-	-
B	42.7	2	0.7	1.1	-	-	-	5	1.0	2.0	-	-	-
C	19.0	-	-	-	-	-	-	2	0.4	0.4	-	-	-
D	26.8	8	2.9	2.7	15	10.1	10.1	2	0.4	0.5	-	-	-
E	13.8	-	-	-	1	0.7	0.3	2	0.4	0.3	-	-	-
F	20.2	2	0.7	0.5	39	26.2	19.6	-	-	-	412	100	100
G	39.0	3	1.1	1.5	6	4.0	5.8	-	-	-	-	-	-
H	19.7	74	27.1	18.1	17	11.4	8.3	-	-	-	-	-	-
I	48.4	63	23.1	37.8	12	8.1	14.5	-	-	-	-	-	-
J	18.3	5	1.8	1.1	7	4.7	3.2	-	-	-	-	-	-
K	38.0	8	2.9	3.8	5	3.4	4.7	-	-	-	-	-	-
L	26.3	50	18.3	16.3	22	14.8	14.4	-	-	-	-	-	-
M	29.8	6	2.2	2.2	17	11.4	12.6	-	-	-	-	-	-
N	30.0	8	2.9	3.0	3	2.0	2.2	-	-	-	-	-	-
O	35.1	6	2.2	2.6	4	2.7	3.5	-	-	-	-	-	-
P	27.1	1	0.4	0.3	-	-	-	-	-	-	-	-	-
Total		273	100	100	149	100	100	523	100	100	412	100	100

Table S6. Protein content of the pollen lysate estimated using the Bradford assay for three replicates per diet. SD = standard deviation

		Protein Content mg/mL				
		1	2	3	Mean	SD
ER 1	Polyfloral	32.290	29.102	37.408	32.933	4.190
	Monofloral	55.277	58.297	64.505	59.360	4.705
ER 2	Polyfloral	39.337	39.421	45.881	41.546	3.754
	Monofloral	58.129	58.465	72.978	63.191	8.478

Table S7. Statistical outputs from linear mixed effects models (LMER) in R for comparing consumption between monofloral (intercept) and polyfloral colonies. **a)** total mass of pollen consumed by colonies per week, **b)** total volume of sucrose solution consumed by colonies per week **c)** mass of pollen consumed per gram of colony mass per week, and **d)** sucrose consumed per gram of colony mass per week. For **a)** and **b)**, a 2nd order polynomial relationship best fitted the data. Asterisks highlighting significant differences (alpha values: 0.05 * 0.01 ** 0.001 ***).

a) LMER (pollen consumption ~ diet * poly(week,2) + ER + (week|colony))						
	**Estimate**	**S.E.**	**df**	**t value**	**P value**	
(Intercept)	9.583	0.454	22.462	21.111	2.62e-16	***
dietpoly	-0.160	0.548	19.714	-0.293	0.773	
poly(week, 2) 1	21.325	2.735	21.993	7.798	9.02-e08	***
poly(week, 2) 2	-11.159	2.006	94.000	-5.562	2.49e-07	***
ER1	-1.765	0.474	21.000	-3.725	0.001	**
dietpoly:poly(week,2) 1	-1.735	3.867	21.993	-0.449	0.658	
dietpoly:poly(week,2) 2	0.125	2.837	94.000	0.044	0.965	

b) LMER (sucrose consumption ~ diet * poly(week,2) + ER + (week|colony))						
	**Estimate**	**S.E.**	**df**	**t value**	**P value**	
(Intercept)	169.950	4.289	21.587	39.628	<2e-16	***
dietpoly	-20.239	5.165	18.552	-3.918	0.001	***
poly(week,2)1	128.272	35.922	21.999	3.571	0.002	**
poly(week,2)2	-109.746	24.165	93.998	-4.541	1.66e-05	***
ER1	-46.161	4.496	21.000	-10.267	1.22e-09	***
dietpoly:poly(week,2)1	-95.765	50.801	21.999	-1.885	0.073	
dietpoly:poly(week,2)2	32.904	34.175	93.998	0.963	0.338	

c) LMER (pollen consumed per gram colony mass ~ diet * week + (week|colony))						
	**Estimate**	**S.E.**	**df**	**t value**	**P value**	
(Intercept)	1.036	0.307	22.012	3.376	0.003	**
dietpoly	0.079	0.434	22.012	0.181	0.858	
week	-0.105	0.037	22.010	-2.794	0.011	*
dietpoly:week	-0.007	0.053	22.010	-0.125	0.902	

d) LMER (sucrose consumed per gram colony mass ~ diet * week + (week|colony))						
	**Estimate**	**S.E.**	**df**	**t value**	**P value**	
(Intercept)	2.733	0.103	22.000	26.636	<2e-16	***
dietpoly	-0.109	0.145	22.000	-0.752	0.460	
week	-0.198	0.024	22.000	-8.210	3.83e-08	***
dietpoly:week	0.017	0.034	22.000	0.510	0.615	

Table S8. Statistical output from a linear mixed effects model (LMER) in R comparing cumulative increase in colony mass between monofloral (intercept) and polyfloral colonies. A 2nd order polynomial relationship best fitted the data, and asterisks highlight significant differences (alpha values: 0.05 * 0.01 ** 0.001 ***).

LMER (cumulative colony mass ~ diet * poly(week,2) + ER + (week|colony))						
	**Estimate**	**S.E.**	**df**	**t value**	**P value**	
(Intercept)	81.140	3.435	20.848	23.625	<2e-16	***
dietpoly	-6.354	4.189	17.111	-1.517	0.148	
poly(week,2)1	250.121	19.427	22.000	12.875	1.02e-11	***
poly(week,2)2	23.402	6.494	118.001	3.604	0.000	***
ER1	-18.320	3.478	20.998	-5.268	3.19e-05	***
dietpoly:poly(week,2)1	-88.658	27.474	22.000	-3.227	0.004	**
dietpoly:poly(week,2)2	13.262	9.183	118.001	1.444	0.151	

Table S9. Statistical output from a generalised linear mixed effects model (GLMER) in R comparing cumulative increase in worker production (reared) between monofloral (intercept) and polyfloral colonies **a)** excluding starting pupae number and **b)** including starting pupae number as a fixed factor in the model. A 2nd order polynomial relationship best fitted the data, and asterisks highlight significant differences (alpha values: 0.05 * 0.01 ** 0.001 ***).

For model b), we included starting pupae number to account for polyfloral colonies arriving with a significantly lower number of pupae. However, running the model presented convergence warnings. Furthermore, it did not significantly alter the results of the model, and so we presented only model a) in the main text.

a) GLMER (colony_worker_growth ~ diet * poly(week,2) + ER + (week|colony), family = Poisson)					
	**Estimate**	**S.E.**	**z value**	**P value**	
(Intercept)	4.740	0.107	44.395	<2e-16	***
dietpoly	-0.199	0.122	-1.635	0.102	
poly(week,2)1	6.614	0.475	13.926	<2e-16	***
poly(week,2)2	-1.243	0.149	-8.328	<2e-16	***
ER1	-0.429	0.127	-3.389	0.001	***
dietpoly:poly(week,2)1	-0.122	0.678	-0.180	0.857	
dietpoly:poly(week,2)2	-0.052	0.219	-0.236	0.813	

b) GLMER (colony_worker_growth ~ diet * poly(week,2) + ER + starting pupae number + (week|colony), family = Poisson)					
	**Estimate**	**S.E.**	**z value**	**P value**	
(Intercept)	4.072	0.184	22.121	<2e-16	***
dietpoly	-0.098	0.097	-1.013	0.311	
poly(week,2)1	6.615	0.472	14.009	<2e-16	***
poly(week,2)2	-1.244	0.149	-8.338	<2e-16	***
ER1	-0.311	0.101	-3.080	0.002	**
starting pupae number	0.016	0.004	4.057	4.97e-05	***
dietpoly:poly(week,2)1	-0.157	0.674	-0.233	0.816	
dietpoly:poly(week,2)2	-0.047	0.219	-0.216	0.829	

Table S10. Statistical outputs from generalised linear models (GLM) in R when analysing the difference in total number of a) and b) workers and c) pupae by the end of the experiment for colonies assigned to monofloral (intercept) and polyfloral diet. Asterisks highlight significant differences (alpha values: 0.05 * 0.01 ** 0.001 ***).

For model b), we again included starting pupae number in the model analysing total worker production, in order to account for polyfloral colonies arriving with a significantly lower number of pupae. Including starting pupae number did not appear to affect the result of the models, and so we presented only model a) in the main text.

Note that model c) excludes data for Colony D, which had begun producing gyne pupae (~600 mg) and was therefore not included in the analysis.

a) GLM (workers ~ diet + ER, family = Poisson)					
	**Estimate**	**S.E.**	**z value**	**P value**	
(Intercept)	5.372	0.025	215.586	<2e-16	***
dietpoly	-0.192	0.031	-6.170	6.8e-10	***
ER1	-0.253	0.031	-8.135	4.11e-16	***

b) GLM (workers ~ diet + ER + starting pupae number, family = Poisson)					
	**Estimate**	**S.E.**	**z value**	**P value**	
(Intercept)	4.921	0.058	85.451	<2e-16	***
dietpoly	-0.134	0.032	-4.224	2.39e-05	***
ER1	-0.150	0.034	-4.457	8.30e-06	***
starting pupae number	0.010	0.001	8.924	<2e-16	***

c) GLM (pupae ~ diet + ER, family = Poisson)					
	**Estimate**	**S.E.**	**z value**	**P value**	
(Intercept)	4.437	0.042	106.994	<2e-16	***
dietpoly	-0.189	0.056	-3.385	0.001	***
ER1	-0.832	0.061	-13.556	<2e-16	***

Table S11. Statistical output from a linear mixed effects model (LMER) in R comparing change in worker mass over the course of the experiment between monofloral (intercept) and polyfloral colonies. A 2nd order polynomial relationship best fitted the data, and asterisks highlight significant differences (alpha values: 0.05 * 0.01 ** 0.001 ***).

LMER (worker mass ~ diet * poly(week,2) + ER + (week|colony))						
	**Estimate**	**S.E.**	**df**	**t value**	**P value**	
(Intercept)	130.653	5.526	19.849	23.644	5.21e-16	***
dietpoly	-10.105	6.351	19.620	-1.591	0.128	
poly(week,2)1	-814.121	190.881	21.758	-4.265	0.000	***
poly(week,2)2	789.219	48.318	3334.620	16.334	<2e-16	***
ER1	15.340	6.356	19.636	2.413	0.026	*
dietpoly:poly(week,2)1	-728.213	270.166	22.087	-2.695	0.013	*
dietpoly:poly(week,2)2	37.307	73.800	3341.534	0.506	0.613	

Table S12. Statistical outputs from generalised linear models (GLM) in R when analysing the difference in total mortality of workers by the end of the experiment between monofloral (intercept) and polyfloral diet for colonies that included (top three rows) and excluded (lower three rows) the two colonies (one monofloral, one polyfloral) experiencing >50% mortality. Asterisks highlight significant differences (alpha values: 0.05 * 0.01 ** 0.001 ***).

GLM (cbind (total mortality, total workers alive at end) ~ diet + ER, family = binomial)					
	**Estimate**	**S.E.**	**z value**	**P value**	
(Intercept)	-1.510	0.060	-25.168	<2e-16	***
dietpoly	0.235	0.073	3.202	0.001	**
ER1	-0.219	0.075	-2.917	0.004	**
(Intercept)	-1.738	0.066	-26.239	<2e-16	***
dietpoly	0.220	0.079	2.789	0.005	**
ER1	0.016	0.079	0.207	0.836	

Table S13. Total number of males produced by each colony across experimental replicates 1 and 2.

Experimental replicate	Colony identification	Diet	Total number of males produced
1	A	Polyfloral	0
1	B	Polyfloral	33
1	C	Polyfloral	0
1	D	Polyfloral	0
1	E	Polyfloral	0
1	F	Polyfloral	0
1	G	Monofloral	6
1	H	Monofloral	3
1	I	Monofloral	1
1	J	Monofloral	0
1	K	Monofloral	1
1	L	Monofloral	0
2	AA	Monofloral	3
2	BB	Monofloral	45
2	CC	Monofloral	7
2	DD	Monofloral	108
2	EE	Monofloral	32
2	FF	Monofloral	5
2	GG	Polyfloral	7
2	HH	Polyfloral	4
2	II	Polyfloral	1
2	JJ	Polyfloral	1
2	KK	Polyfloral	47
2	LL	Polyfloral	15

Table S14. Mean (± s.d.) number of larvae and pupae found per colony on dissection at the end of the experiment, as well as mass (g) of the nest structure once all brood, adult workers and the queen had been removed.

	Monofloral diet	Polyfloral diet
Small larvae	26.3 ± 7.0	19.4 ± 4.3
Medium larvae	28.8 ± 9.0	29.3 ± 4.9
Large larvae	5.3 ± 1.3	5.3 ± 1.5
Pupae	60.7 ± 11.2	49.3 ± 9.3
Nest structure (g)	20.6 ± 1.6	21.1 ± 2.4

Table S15. Statistical outputs from linear mixed effects models (LMER) in R when analysing **a)** male mass and **b)** average pupal mass per colony dissected from the nest, at the end of the experiment between monofloral (intercept) and polyfloral colonies. For male mass the data was log10 transformed to better meet the assumptions of normality. For the pupal mass, one polyfloral colony was not considered in this analysis because it was producing gyne pupae (~600mg) at the end of the experiment. Asterisks highlight significant differences (alpha values: 0.05 * 0.01 ** 0.001 ***).

a) LMER (male mass ~ diet + ER + (1|colony))						
	**Estimate**	**S.E.**	**df**	**t value**	**P value**	
(Intercept)	2.326	0.039	8.727	59.673	1.05e-12	***
dietpoly	-0.113	0.054	9.607	-2.103	0.063	
ER1	-0.022	0.064	10.975	-0.344	0.737	

b) LM (pupa mean mass ~ diet + ER)					
	**Estimate**	**S.E.**	**t value**	**P value**	
(Intercept)	334.24	25.65	13.032	3.12e-11	***
dietpoly	-73.48	30.08	-2.410	0.026	*
ER1	19.73	30.08	0.656	0.519	
